# Poorly Controlled New-Onset Diabetes Mellitus and Other Atypical Signs as an Early Sign of Pancreatic Adenocarcinoma

**DOI:** 10.7759/cureus.62319

**Published:** 2024-06-13

**Authors:** Francisco Josué Cordero Pérez, Pablo Rodríguez López, Paula Oleaga Gómez, Marta Antona Herranz, Eva Purificación Martín Garrido

**Affiliations:** 1 Department of Internal Medicine, Complejo Asistencial de Zamora, Zamora, ESP; 2 Department of Medicine, Faculty of Medicine, University of Salamanca, Salamanca, ESP; 3 Department of Radiodiagnostics, Complejo Asistencial de Zamora, Zamora, ESP; 4 Department of Gastroenterology, Complejo Asistencial de Zamora, Zamora, ESP

**Keywords:** new-onset diabetes, acute pancreatitis (ap), acute pancreatitis complications, pancreatic pseudocyst (ppc), adenocarcinoma pancreas

## Abstract

A 50-year-old man presented with poorly controlled new-onset diabetes mellitus. Six months after diagnosis, episodes of intense abdominal pain with vomiting appeared. Abdominal CT revealed signs of acute pancreatitis with structural changes in the pseudocysts. In the absence of biliary lithiasis or a toxic etiology of acute pancreatitis, the patient progressed unfavorably with increased abdominal pain and fever. Control imaging tests (two and 10 months later) showed the evolution of phlegmonous/necrotic collections, together with portal vein thrombosis and splenomegaly. Given the suggestive signs of possible occult malignancy, such as portal thrombosis, histological analysis of the ascitic fluid revealed a pancreatic adenocarcinoma. Despite the initiation of chemotherapy, the patient died 12 months after diagnosis.

## Introduction

Pancreatic ductal adenocarcinoma (PDAC) presents significant challenges in terms of its diagnosis and treatment. It is the most common type of pancreatic tumor and the fourth leading cause of cancer-related mortalities worldwide. A typical presentation consists of a hypovascular mass with dilation of the upstream pancreatic duct and abrupt cutting of the duct due to abdominal pain, jaundice, and weight loss [1]. This case report focuses on a 50-year-old man with newly diagnosed and poorly controlled diabetes mellitus (DM) and episodes of acute pancreatitis with structural changes and pseudocysts six months before the diagnosis of pancreatic adenocarcinoma. Imaging and histopathological analyses were also performed.

The association between PDAC and new-onset diabetes is well-documented, with approximately 40% of PDAC cases presenting with this condition. However, the exact temporal relationship between the onset of diabetes and PDAC diagnosis remains unclear [2,3]. PDAC can exhibit atypical imaging features that overlap with other pancreatic abnormalities, further complicating the diagnostic process. In this context, pancreatic pseudocysts can be associated with both pancreatitis and malignancy, highlighting the importance of a thorough evaluation [4-7].

This case highlights the need to consider the possibility of an underlying malignant lesion in the presence of significant pancreatic alterations, especially in patients with atypical symptoms and risk factors such as newly diagnosed DM. Early detection and management of such cases are crucial for improving clinical outcomes and patient quality of life [4-8].

## Case presentation

A 50-year-old male was admitted to our hospital with a history of poorly controlled DM under endocrinological follow-up that was diagnosed one year ago, without a family history of diabetes. He presented to the emergency department (referred to in his endocrinology consultations) with severe upper abdominal pain, unremarkable blood work, and no elevation in pancreatic enzymes. An abdominal CT scan revealed peripancreatic cystic lesions of varying sizes suggestive of pseudocysts, likely due to episodes of previous pancreatitis. The largest measured 11.6 cm in the left hypochondrium, and an 8.2 cm lesion was noted at the anterior pancreas, along with smaller lesions in the pancreatic tail, indicative of recurrent pancreatitis. Additionally, portal vein thrombosis involving the right and left branches and extrahepatic regions, along with free gastrohepatic and pelvic fluid and homogeneous splenomegaly measuring 14.7 cm, were observed. The patient was admitted to the Department of Gastroenterology for further evaluation.

**Figure 1:**
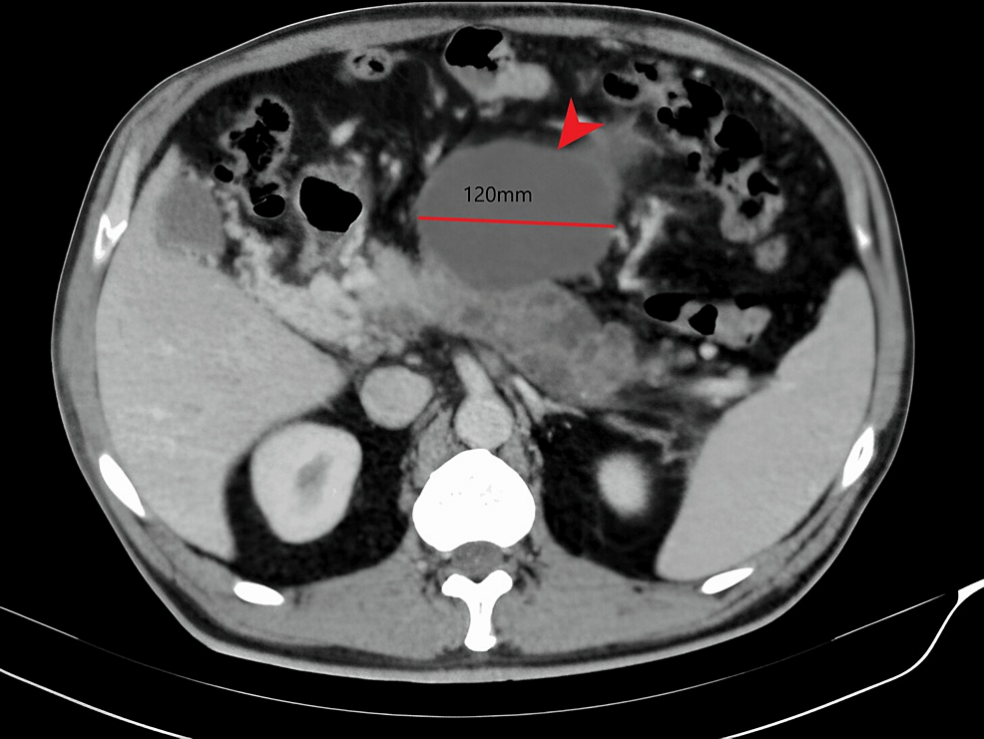
Initial CT scan It shows portal thrombosis right and left branches in the hepatic hilum, probably secondary to thrombosis during the time of evolution. In the pancreas, two well-defined anechoic structures are identified with echogenic content in its interior of 12 cm and another of 9 cm suggestive of pancreatic pseudocysts that prevent the complete visualization of the gland. The body of the pancreas is also partially visualized with multiple chiascopic images between 12 and 15 mm.

Upon re-evaluation, the patient reported a sudden episode of abdominal pain in the epigastrium one month earlier, followed by persistent abdominal discomfort. It was suspected to be progressively evolving acute pancreatitis, leading to ultrasound-guided pseudocystogastrostomy. Etiological investigations for acute pancreatitis were negative (no gallstones, toxic substance consumption, metabolic disorders, and autoimmune study). Given the apparent resolution of the pseudocysts on follow-up CT (Figure 1), the decision was made for discharge and outpatient follow-up.

Two months later, the patient was readmitted due to a new episode of abdominal pain and fever, and laboratory findings indicated leukocytosis (14,630 cells/mm³), coagulopathy (INR 5.18, a. protr 12%), glucose level 201, GPT 87, alkaline phosphatase 667, amylase 18, and CRP 261 (Table 1).

**Table 1:** Laboratory values Values ​​in blood analysis throughout the development of the case report ALT: Alanine transaminase; CEA: carcinoembryonic antigen; CRP: C-reactive protein; GGT: gamma-glutamyltransferase

	Eight months before	Day 1	Day 25	Ten months
Hb [13.0-17.0 g/dL]	16.5	13.0	12.4	9.1
Leukocytes [4.00-11.00 x10e3 cells/mm^3^]	7.0	14.63	12.7	5.01
Glucose [74-106 mg/dL]	387	201	136	201
ALT (GPT) [5-41 UI/L]	45	87	62	12
Alkaline Phosphatase [40-130UI/L]	109	667	789	426
GGT [3-60 UI/L]	100	814	1739	455
CRP [0-5.0 mg/dL]	22.6	261	55.4	101.5
HbA1c (%)	11.3	9.7	7.8	7.3
Ca 19.9 [U/mL]	-	-	3500	29007
CEA [μg/L]	-	-	-	32.9

**Figure 2:**
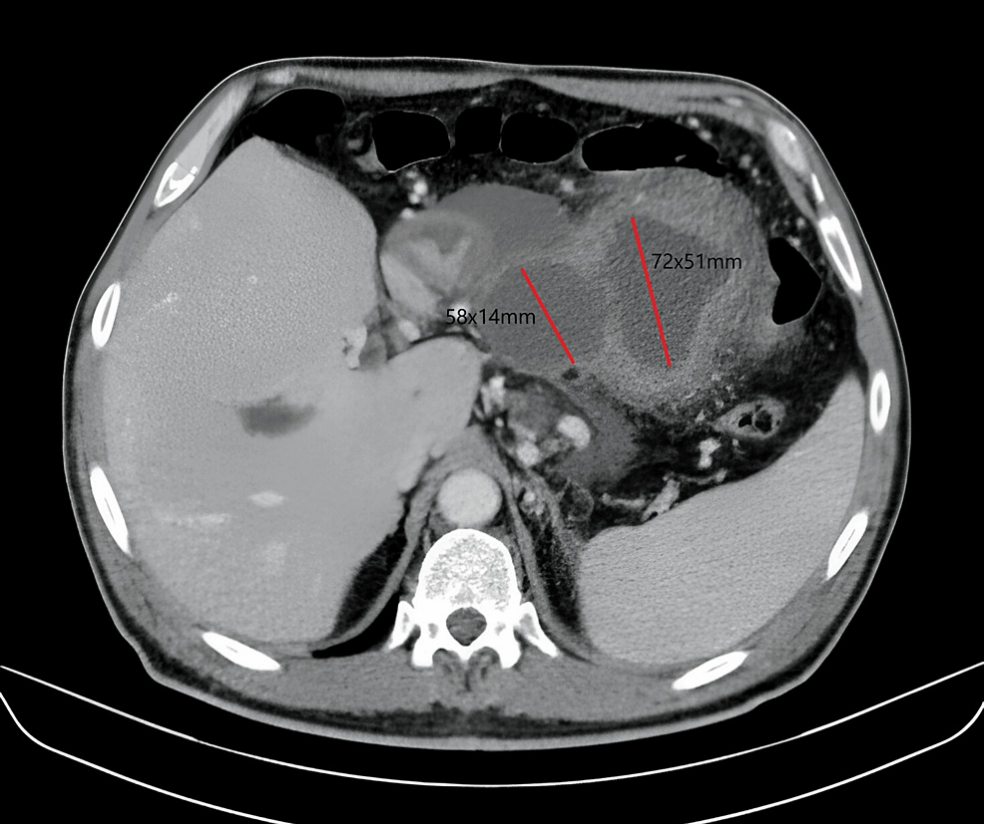
CT scan at two months There is mural thickening of the pseudocyst wall, with possible superinfection. This collection seems to be connected, in a filiform form, with another one of more medial and caudal locations measuring 72x51x56 mm. There are other peripancreatic collections of 58x14x33 mm. and other multiple ones of very small size, which do not allow visualization of the pancreatic body and tail. Wirsung's dilatation is observed in this region of the body and tail, with suspicion of the underlying neoplastic process.

**Figure 3:**
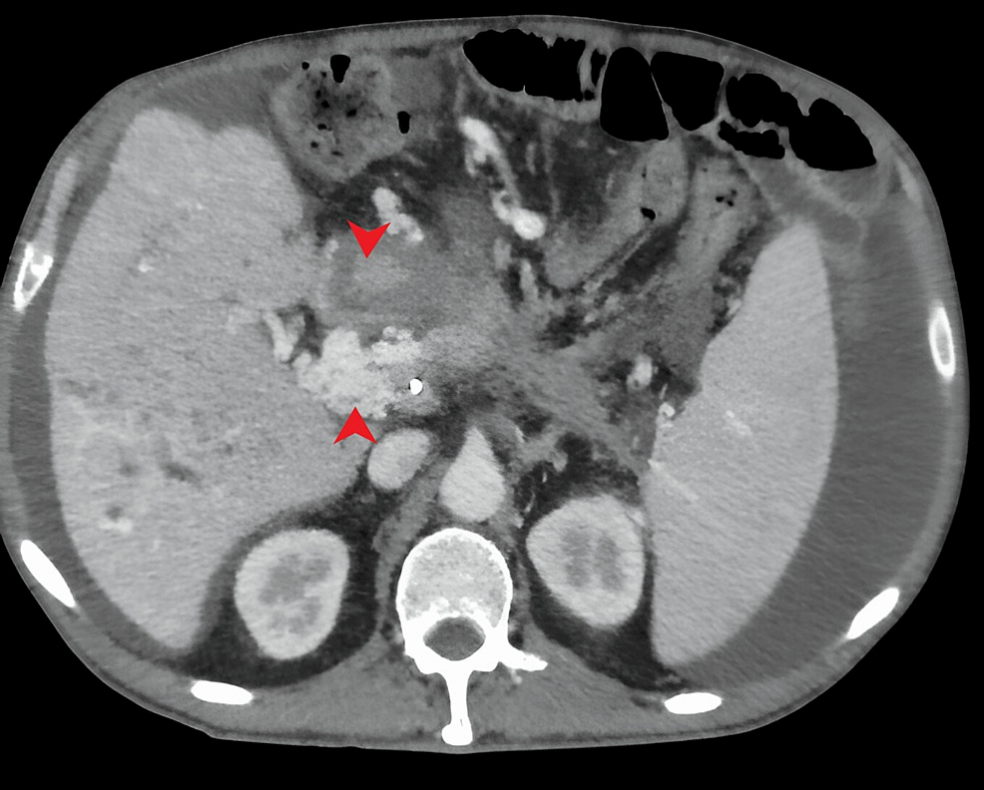
CT scan after 10 months A pancreatic tumor was observed at the level of the intrapancreatic common bile duct, complete destructuring of the pancreas at the level of the head and uncinate process, and multiple hypodense lesions in the left hepatic lobe and upper segments of the right hepatic lobe, in addition to complete thrombosis of the portal and its branches, with significant cavernomatosis/collateral circulation at the level of the hepatic hilum.

A repeat CT scan revealed inflammatory changes in the pancreas with a phlegmonous/necrotic appearance, increased size of previous lesions (now measuring 27 and 30 mm) located between the stomach and the pancreatic body, and a 30 mm lesion caudal to the pancreatic tail. Additionally, multiple small collections within the pancreas; involvement of peripancreatic fat; appearance of ascites with abundant free perihepatic, perisplenic, and intra-abdominal fluid; and complete portal vein thrombosis with cavernomatosis were noted. These findings raised concerns about the possibility of underlying malignancy (Figures 2, 3).

**Figure 4:**
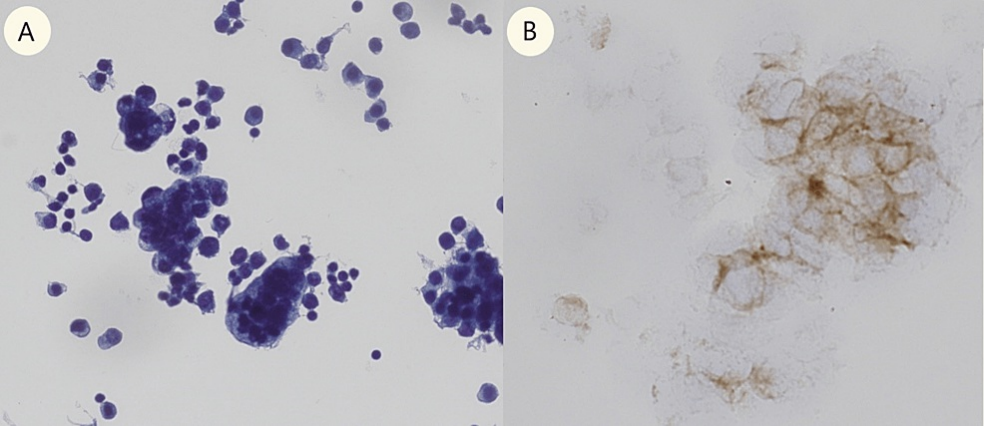
Ascitic fluid cytology A) Cytology showing abundant clusters of cells with an epithelioid morphology. They have enlarged nuclei and irregularities that form three-dimensional groups with anisokaryosis, vacuoles, nuclear overlap, and ample cytoplasm. B) Cells were positive for BerEp4.

In response to these findings, ultrasound-guided paracentesis of ascitic fluid was performed for histopathological analysis, revealing compatibility with pancreatic adenocarcinoma (Figure 4), and a mass compatible with adenocarcinoma was observed in the next CT scan (Figure 3). The patient underwent comprehensive staging evaluation and was referred to the oncology department with a diagnosis of stage IV pancreatic adenocarcinoma. Chemotherapy was initiated, but the patient succumbed to the disease 12 months after the diagnosis.

## Discussion

**Figure 5:**
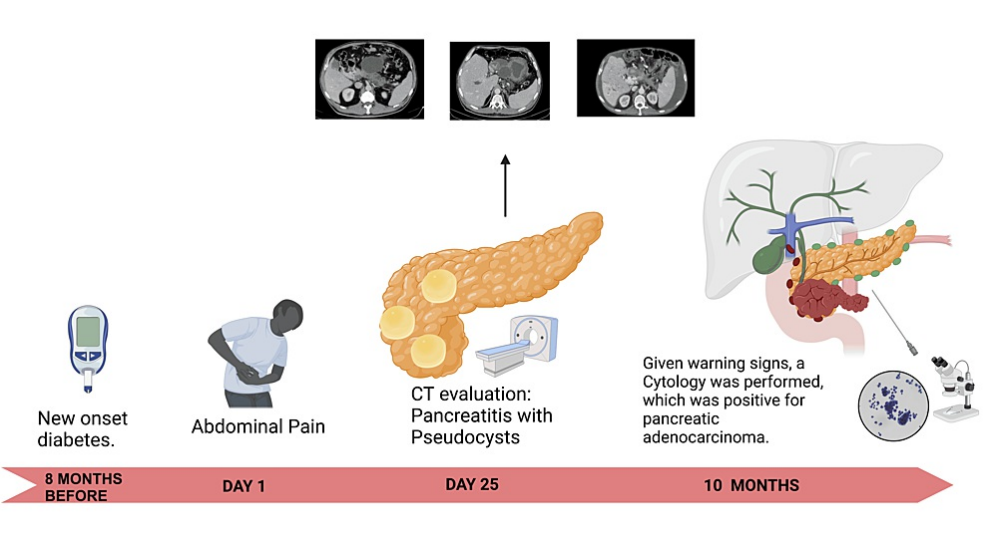
Summary of the case report Clinical evolution: Timeline of the evolution of the case depicting the main events from new-onset diabetes, including symptom onset, complementary examinations, and management. The onset of rapid progression can be appreciated. This is an own elaboration.

PDAC presents a significant challenge in terms of diagnosis and treatment in the field of oncology. Its conventional presentation as a hypovascular infiltrative solid mass often hinders its early detection and effective treatment [1]. In this way, we can understand our case report as pancreatic adenocarcinoma with atypical manifestations (Figure 5).

The early manifestations of PDAC are diverse and may include the onset of diabetes, acute and chronic pancreatitis, and characteristic radiological abnormalities. Among the clinical manifestations, there is a well-documented association between PDAC and new-onset diabetes, with approximately 40% of PDAC cases presenting with this condition. However, the exact temporal relationship between the onset of diabetes and the diagnosis of PDAC remains unclear, with some cases showing a prevalence of diabetes 24 months prior to diagnosis [2,3].

Regarding radiological manifestations, PDAC may exhibit atypical imaging features that can overlap with various other pancreatic abnormalities, including inflammatory conditions such as acute and chronic mass-forming pancreatitis, autoimmune pancreatitis, and paraduodenal pancreatitis, as well as pancreatic neuroendocrine tumors, solid pseudopapillary neoplasms, and metastases. These overlapping features pose significant challenges to the accurate diagnosis of PDAC, often requiring additional investigations for differentiation [4,5].

Additionally, acute pancreatitis can mimic PDAC, presenting with local, interstitial edematous, or necrotizing forms. Studies have indicated an association between idiopathic acute pancreatitis and early manifestations of PDAC, that 6.8%-13.8% of patients with acute pancreatitis have coexisting PDAC, particularly in patients older than 40 years of age (10.7 %). The symptoms of acute pancreatitis, such as abdominal pain, weight loss, and jaundice, can overlap with those of PDAC, further complicating the diagnostic process, pudiendo encontrar elevación de lipasa y amilasa, agrandamiento del pancreas, heterogenecidad e inflamación peripancreatica y liquido libre, asi como ampuntacion del wirsung [4,6].

Cystic formations represent approximately 8% of all presentations in a subset of PDAC cases. These structural changes encompass manifestations such as PDAC of large glands with small cysts (21%), degenerative cystic alterations (11%), PDAC with retention cysts, and those closely associated with pseudocysts due to tumor-associated pancreatitis (5%). Differentiating between benign and malignant cystic tumors, particularly pancreatic pseudocysts, requires careful consideration of clinical history, imaging findings, and tumor markers. Treatment decisions, whether surgical, interventional, or endoscopic, are recommended based on the complications, symptoms, or specific characteristics of the cyst [4,5,7,8].

Recent studies have highlighted the intricate relationship between pancreatic pseudocysts and malignancy, emphasizing the importance of accurate diagnosis and treatment. Although pseudocysts are prevalent in both acute and chronic pancreatitis, their association with malignancy is relatively minor, necessitating thorough evaluation using advanced imaging techniques such as transabdominal ultrasound, computed tomography, magnetic resonance imaging, magnetic resonance cholangiopancreatography, and endoscopic ultrasound (EUS). Fine-needle aspiration guided by EUS has emerged as a fundamental tool for evaluating cystic fluid and tumor markers to differentiate pseudocysts from potentially malignant lesions [5,8].

## Conclusions

In conclusion, we must consider the presence of an underlying malignant lesion in the face of the pancreatic alterations mentioned above. A good approach is to request CA 19-9 levels and evaluate the need for biopsy to perform close follow-up to identify any possible malignant transformation. Therefore, it is necessary to consider that certain benign clinical manifestations may constitute precursors to malignancy and the development of pancreatic adenocarcinoma. This clinical case serves as an example of a case of pancreatic adenocarcinoma that presented atypically with acute pancreatitis with structural alterations (pancreatic pseudocysts), additionally presenting a history of new-onset poorly controlled DM 12 months prior. 

## References

[1] Cho HW, Choi JY, Kim MJ, Park MS, Lim JS, Chung YE, Kim KW (2011). Pancreatic tumors: emphasis on CT findings and pathologic classification. Korean J Radiol.

[2] Roy A, Sahoo J, Kamalanathan S, Naik D, Mohan P, Kalayarasan R (2021). Diabetes and pancreatic cancer: exploring the two-way traffic. World J Gastroenterol.

[3] Chari ST, Leibson CL, Rabe KG, Timmons LJ, Ransom J, de Andrade M, Petersen GM (2008). Pancreatic cancer-associated diabetes mellitus: prevalence and temporal association with diagnosis of cancer. Gastroenterology.

[4] Miller FH, Lopes Vendrami C, Hammond NA, Mittal PK, Nikolaidis P, Jawahar A (2023). Pancreatic cancer and its mimics. Radiographics.

[5] Miller FH, Lopes Vendrami C, Recht HS (2022). Pancreatic cystic lesions and malignancy: assessment, guidelines, and the field defect. Radiographics.

[6] Schima W, Böhm G, Rösch CS, Klaus A, Függer R, Kopf H (2020). Mass-forming pancreatitis versus pancreatic ductal adenocarcinoma: CT and MR imaging for differentiation. Cancer Imaging.

[7] Kosmahl M, Pauser U, Anlauf M, Klöppel G (2005). Pancreatic ductal adenocarcinomas with cystic features: neither rare nor uniform. Mod Pathol.

[8] Bagci P, Andea AA, Basturk O, Jang KT, Erbarut I, Adsay V (2012). Large duct type invasive adenocarcinoma of the pancreas with microcystic and papillary patterns: a potential microscopic mimic of non-invasive ductal neoplasia. Mod Pathol.

